# Genetic Deficiency of GABA Differentially Regulates Respiratory and Non-Respiratory Motor Neuron Development

**DOI:** 10.1371/journal.pone.0056257

**Published:** 2013-02-15

**Authors:** Matthew J. Fogarty, Karen L. Smallcombe, Yuchio Yanagawa, Kunihiko Obata, Mark C. Bellingham, Peter G. Noakes

**Affiliations:** 1 School of Biomedical Sciences, University of Queensland, Brisbane, Queensland, Australia; 2 Queensland Brain Institute, University of Queensland, Brisbane, Queensland, Australia; 3 Gunma University Graduate School of Medicine, Maebashi, Japan; 4 Japan Science and Technology Agency, CREST, Tokyo, Japan; 5 Obata Research Unit, RIKEN Brain Science Institute, Saitama, Japan; University of Edinburgh, United Kingdom

## Abstract

Central nervous system GABAergic and glycinergic synaptic activity switches from postsynaptic excitation to inhibition during the stage when motor neuron numbers are being reduced, and when synaptic connections are being established onto and by motor neurons. In mice this occurs between embryonic (**E**) day 13 and birth (postnatal day 0). Our previous work on mice lacking glycinergic transmission suggested that altered motor neuron activity levels correspondingly regulated motor neuron survival and muscle innervation for all respiratory and non respiratory motor neuron pools, during this period of development [1]. To determine if GABAergic transmission plays a similar role, we quantified motor neuron number and the extent of muscle innervation in four distinct regions of the brain stem and spinal cord; hypoglossal, phrenic, brachial and lumbar motor pools, in mice lacking the enzyme GAD67. These mice display a 90% drop in CNS GABA levels ( [2]; this study). For respiratory-based motor neurons (hypoglossal and phrenic motor pools), we have observed significant drops in motor neuron number (17% decline for hypoglossal and 23% decline for phrenic) and muscle innervations (55% decrease). By contrast for non-respiratory motor neurons of the brachial lateral motor column, we have observed an increase in motor neuron number (43% increase) and muscle innervations (99% increase); however for more caudally located motor neurons within the lumbar lateral motor column, we observed no change in either neuron number or muscle innervation. These results show in mice lacking physiological levels of GABA, there are distinct regional changes in motor neuron number and muscle innervation, which appear to be linked to their physiological function and to their rostral-caudal position within the developing spinal cord. Our results also suggest that for more caudal (lumbar) regions of the spinal cord, the effect of GABA is less influential on motor neuron development compared to that of glycine.

## Introduction

Neuronal cell death is a necessary process that is essential for the developmental refinement of complex neural networks. In the neuromotor system of the mouse, over 50% of motor neurons die between embryonic day [**E**] 13 and birth [1], [3], [4], [5], [6], [7]. The number of motor neurons lost depends on the amount of synaptic activity in the developing neuromuscular pathway, which in turn controls the level of muscle activity. When muscle activity is experimentally reduced, more motor neurons survive. Motor axons branch more under these conditions, and this is thought to increase their access to target-derived trophic factors, thereby increasing survival [5], [6], [7], [8], [9], [10]. By contrast, interventions that increase motor neuron activity and/or muscle activity lead to reductions in muscle nerve branching and neuromuscular synapse number. Fewer motor neurons survive through the cell death period when muscle activity is experimentally increased, possibly due to reduced access to trophic factors resulting from fewer formed neuromuscular synapses [1], [11]. These observations have led to the idea that muscle electrical activity evoked by neuromuscular synaptic activity is the regulator of motor neuron numbers during developmental cell death. This is thought to constitute an intrinsic safety mechanism that adjusts the number of surviving motor neurons innervating a given muscle to the needs of the muscle for complete and effective control at birth [6], [12], [13], [14], [15].

This normal loss of motor neurons during development overlaps with the period when they first receive synaptic connections from other neurons (central synapses; [14], [16], [17], [18]), and when they form their output synapses on muscle cells (neuromuscular synapses; [19], [20], [21]). Initially all synaptic inputs onto motor neurons are excitatory, including glycinergic and GABAergic inputs [22], [23], [24], [25]. Later in development, glycinergic and GABAergic inputs become inhibitory [23], [24], [25], [26]. This suggests that glycinergic and GABAergic transmission could play a role in motor neuron development.

So far, our studies have shown that central glycinergic transmission does play an essential role in these processes [1]. In mutant mice lacking gephyrin, a cytoplasmic molecule that is needed for the post-synaptic clustering of glycine receptor clusters [27], [28], [29] and therefore a model of perturbed glycinergic transmission, motor neuron number and muscle nerve branching are altered during the period of neuronal cell death. The nature of these alterations depended upon the motor nuclei studied. Respiratory motor neurons (hypoglossal and phrenic motor pools) displayed decreased motor neuron survival and decreased innervation of their target respiratory muscles. By contrast, limb-innervating lumbar motor neurons showed increased neuronal survival and increased innervation of the leg muscles [1]. Gephyrin can also cause the postsynaptic clustering of some GABA_A_ receptors [28], [30], [31], [32], [33], however our pharmacological studies on wild type and gephyrin-deficient mice, where we compared motor neuron activity responses to glycine or GABA suggested that the loss of glycinergic transmission in gephyrin deficient mice was the primary disturbance [1].

In the present study, we have used mice lacking the 67 kDa isoform of glutamate decarboxylase (GAD67), to investigate the role of GABA in motor neuron development. This isoform of GAD is responsible for 80 to 90% production of GABA from glutamate in GABAergic neurons during development [2], [34]. In these mutant mice that lack physiological levels of GABA, we have observed significant changes in motor neuron number and the extent of muscle innervation for respiratory and non-respiratory motor neurons, with the exception of motor neurons within the lumbar lateral motor column. These results suggest that GABA has a similar, but more restricted role to that of glycine, in motor neuron development at times when motor neurons are regulating their final numbers within the brain stem and spinal cord.

## Materials and Methods

### Ethics Statement

All experimental procedures were approved by the University of Queensland Animal Ethics Committee (Permit Numbers: 227–09, 924–08, and 152–12), and complied with the policies and regulations regarding animal experimentation and other ethical matters [35]. They were conducted in accordance with the Queensland Government Animal Research Act 2001, associated Animal Care and Protection Regulations (2002 and 2008), the Australian Code of Practice for the Care and Use of Animals for Scientific Purposes, 7th Edition (National Health and Medical Research Council, 2004).

### Mice

Wild type and homozygous GAD67-GFP knock-in mice [2], [34] were used in this study. GAD67-GFP knock-in mice were made by gene targeting green fluorescent protein (GFP) to the locus of the GAD67 gene, enabling GFP expression to be under the control of the endogenous GAD67 promoter [34], and effectively deleting the transcription of endogenous GAD67 (i.e. a knockout of GAD67). Therefore, in this study we refer to heterozygote and homozygous GAD67-GFP knock-in mice as GAD67^+/−^ and GAD67^−/−^ mice respectively. GAD67^+/−^ males and females were time mated, and the observation of a vaginal plug was determined as embryonic day 0.5 (E0.5). Pregnant GAD67^+/−^ females at the appropriate gestational age were sacrificed by cervical dislocation and embryos were harvested by caesarean section. Embryonic mice of both sexes carrying either two normal copies of GAD67 (wild-type, GAD67^+/+^) or two mutant copies of the GAD67 gene (GAD67^−/−^; [34]) were then processed and analyzed as detailed below. Genotype of each embryo used was identified by DNA tail assay as described previously [34].

### Motor Neuron Counts

Brain stem and spinal cords from embryos at embryonic (E) days 13.5, 15.5 and 18.5 (E13.5, E15.5, E18.5) were isolated and then processed for paraffin histology using previously established methods [1]. Transverse serial sections (6–12 µm) were cut in a rostro-caudal direction, stained with thionin in 0.1% (v/v) acetic acid buffer solution. Motor neuron numbers were quantified ipsilaterally in four different motor neuron pool regions, using previously established methods [5], [7], [36]. The hypoglossal (XII) nuclei were identified by anatomical landmarks; the base of the 4^th^ ventricle and central canal of the brain stem. The phrenic lateral motor column (LMC) extends from the 3^rd^ cervical dorsal root ganglia (DRG) to the 5^th^ cervical DRG ganglion. The brachial lateral motor column (LMC) extends from the 6^th^ cervical DRG to the 1^st^ thoracic DRG ganglion, and the lumbar LMC extends from the 2^nd^ lumbar DRG to the 6^th^ lumbar DRG. Ventral horn thickening, LMC enlargement, and the presence of DRG identified the commencement and termination of these motor columns. Landmark identification was aided by the use of mouse brain atlases [37], [38]. To qualify for counting, motor neurons needed to meet the following visual criteria: be large in size; have a darkly stained cytoplasm; a distinct pale nucleus; and a darkly stained nucleolus [36].

In addition, we examined the staining overlap of thionin stained neurons within these motor regions to anti-vesicular acetylcholine transporter (VAChT) antibody staining on adjacent serial sections. Briefly, dissected E18.5 lumbar spinal cords were fixed in 4% paraformaldehyde in phosphate buffered saline pH 7.4 (PBS), followed by washing in PBS and infiltration in 15% sucrose in PBS followed by 30% sucrose in PBS until the tissue had sunk. Tissue was then frozen on dry ice in OCT compound (VWR International, Leuven, Belgium). Frozen blocks were then before cryo-sectioned at 12 µm. Two series of every 10^th^ section were generated, one series was washed in PBS and stained with 0.1% thionin and mounted. The alternate series was washed in PBS, bathed in 2% bovine serum albumin (BSA), 0.1% Triton X-100 in PBS (blocking solution) for 4 hours at 4°C. These sections were then incubated with human anti-mouse VAChT (1∶400 in blocking solution; Clone N6/38, NeuroMab, UC Davis/NIH NeuroMab Facility, CA, USA) overnight at 4°C. Sections were then washed in PBS and incubated with Cy3 conjugated Goat anti-mouse Cy3 (1∶500 in blocking solution; Invitrogen, Victoria, Australia) for 12 hours at 4°C. Sections were then rinsed in PBS and cover slipped in anti-fade mounting media (Bio-Rad, Hercules, CA, USA). Negative control for anti-VAChT staining included omission of the anti-VAChT and substitution with normal mouse IgG at the same concentration as the anti-VAChT antibody.

To ensure systematic random sampling, the initial tissue section to be counted was randomly chosen from the first 5–10 sections at the commencement of the motor neuron pool. To quantify total motor neuron number, every 10^th^ (LMC) or 5^th^ (XII) section of the motor neuron pool was counted. This number was then divided by the number of sections counted and further multiplied by the total number of sections containing the motor neuron pool, as detailed previously [5], [36]. At least one mouse in each age group and genotype was counted on both sides to ensure there were no discrepancies in motor neuron numbers between the left and right LMCs. The mouse genotype was not made available to the researchers conducting the counts (KLS and MJF), until the counts were completed (i.e. counts were performed blind).

### Motor Neuron Volume and Lateral Motor Column Volume

Histological slides used for counting motor neurons were also used to quantify neuronal volume and lateral motor column volumes. For cell volumes, motor neurons within the selected motor neuron pool were chosen by systematic random sampling of every 10^th^ section. The optical fractionator (Stereo Investigator, MBF Biosystems USA) with a counting frame of 100 µm×100 µm and overlaid grid size of 150 µm×150 µm with 3 nucleator rays was used to estimate motor neuron volume [39]. A minimum of 50 motor neurons per motor neuron pool was quantified using the nucleator method with the following formula:
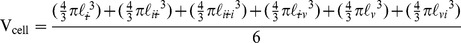
Where is the distance from the centre of the nucleator probe to the edge of the cell soma [39]. For motor pools size, a Cavalieri volume estimate was obtained by using the following formula *V = ∑P • a(p)•t*, where *∑P* is the total number of test points overlaying the motor pool, *a(p)* is the area associated with each grid point (2500 µm) and *t* is the distance between each section (220 µm) [39].

### Limitations of Our Study

**Figure 1:**
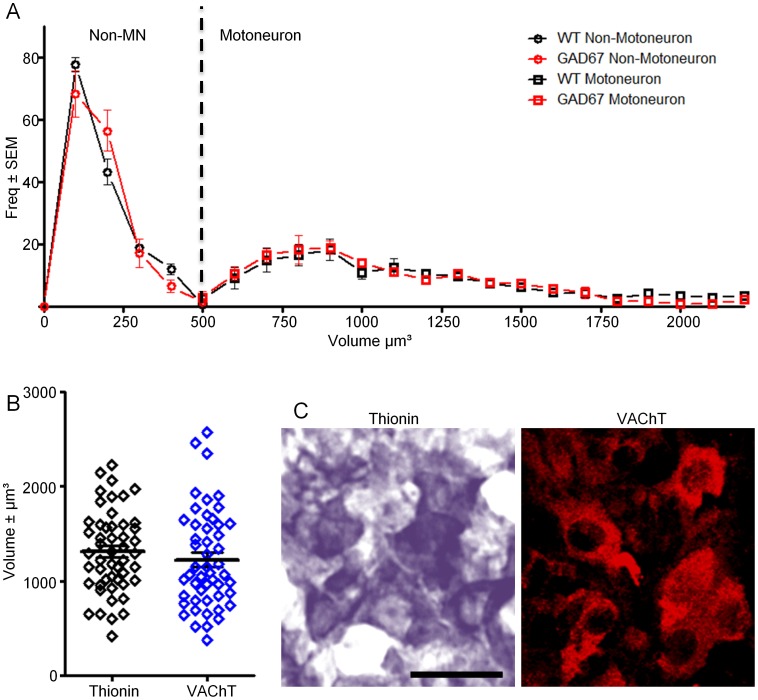
Cell size does not change for motor neurons and non-motor neurons within motor pool regions of GAD67-deficient and wild type mice at E18.5. A shows the mean cell volume frequency histogram for combined hypoglossal, brachial and lumbar motor pools for GAD67-deficient (red lines, GAD67) and wild type litter mates (black lines, WT), at E18.5. There was no shift in cell size across the two genotypes, and each genotype revealed a discrete population of small cells (black and red circles) separated from a broad population of large cells (black and red squares). B shows that volumes of the broad population of large cells from serial sections of a wild-type lumbar spinal cord are positive for both thionin (black diamonds) and VAChT (blue diamonds), demonstrating that the large cells within these motor pools are cholinergic, and by their location are motor neurons. C shows serial sections of the lumbar lateral motor column stained for thionin and VAChT, from an E18.5 wild type mouse. Note all large cells within this motor region are positive for thionin and VAChT. Scale bar for C = 25 µm.

In our study we have used accepted histological criteria to identify motor neurons; namely location, size (α- motor neurons are large when compared to other surrounding cells, such as interneurons and γ- motor neurons), large cytoplasm (all surrounding cells including interneurons have very little observable cytoplasm), and the presence of a prominent nucleolus [1], [5], [7], [36]. These criteria assume that there is very little change in motor neuron size in mice lacking GABA, and that all large cells within the motor pools are motor neurons. To address these two assumptions and add support to our criteria, we have measured and compared the volumes of all cells located within motor regions of the spinal cord (ventral horn) and brain stem (hypoglossal) from GAD67-deficient and wild type littermate mice at E18.5. We observed no shift in the distribution of large or small cells within these regions (Figure 1A). Next, we have stained these motor regions with thionin and the adjacent serial section with anti-VAChT (an antibody that positively identifies cholinergic neurons, [40], [41]), of E18.5 wild type mice. All large cells within these motor regions stain for both thionin and VAChT (Figure 1B and 1C). Together this data supports our assumption that all large thionin positive neurons within these motor regions are cholinergic, and that there is no shift in cell size within these motor pools across the two genotypes.

In respect to the surrounding anatomical landmarks that helps define a motor pool, it is possible that these may have shifted between animal and across genotype, complicating our comparisons. We believe we addressed this potential pitfall by serially sectioning the entire brain stem and spinal cord allowing us to successfully locate the beginning and end of these motor regions. We did not observe any gross anatomical disturbances in either the brain stem or spinal cord, indicating that any anatomical variations of surrounding structures was minimal. Finally any potential pitfalls in the estimations of neuronal counts, such as double counting or underestimation of counts, have been taken into account as we employ unbiased uniform random sampling for the selection of sections to count, and stereological techniques and formulae to estimate the total numbers of motor neurons within these regions. This approach is the benchmark for estimating the total number of cells within a defined anatomical boundary [39], [42], [43], [44], [45].

### Muscle-nerve Branching

Embryonic diaphragm (innervated by phrenic motor neurons), latissimus dorsi (innervated by brachial motor neurons) and gluteus maximus (innervated by lumbar motor neurons) muscles at E15.5 and E18.5 were processed for whole-mount immunohistochemistry using previously established methods [1], [7]. In brief, embryos were sacrificed and the relevant muscle tissue dissected and immediately fixed in 4% paraformaldehyde in PBS for 30 minutes at room temperature. The muscles were washed once in PBS, and then in PBS containing 0.1 M glycine for 1 hour at room temperature. Post-synaptic acetylcholine receptors (AChRs) were localized using tetramethylrhodamine isothiocyanate (TRITC)-conjugated α-bungarotoxin (1∶400; Sigma, St. Louis MO, USA). Muscles were then blocked in 4% bovine serum albumin and 0.2% Triton X-100 in PBS for 4 hours overnight at 4°C. Next, motor neuron axonal branches were localized by overnight incubation (4°C) with a combination of anti-neurofilament (1∶200; Sigma) and anti-synapsyn I (1∶50; Sigma) primary rabbit antibody mixture in 2% BSA, 0.1% Triton X-100 in PBS. The muscles were then washed 3 times in PBS before the tissue was probed with Alexa 488-conjugated rabbit secondary antibody (1∶500; Invitrogen, Victoria, Australia), for 4 hours at room temperature. The tissue was then washed 3 times in PBS before mounting onto microscope slides with anti-fade mounting media (Bio-Rad). Muscle-nerve branching and neuromuscular synapse number were quantified at the proximal muscle nerve entry point for the gluteus maximus muscle, the distal terminal region of the latissimus dorsi muscle, and at the ventral region of the left hemi-diaphragm (sterno-costal portion) for the diaphragm muscle. Utilizing previously established methods, both the diaphragm and gluteus maximus muscles were quantified for: a) branch extension from the main muscle nerve trunk; b) the number of branches exiting this nerve trunk; and c) the number of peripheral branch bifurcations [1], [7]. The number of neuromuscular synapses, which is defined as the number of AChR clusters that co-localized with neurofilament/synaptophysin immunostaining were also counted [1], [7], [19].

### GABA Quantification

The GABA levels within the cervical and lumbar spinal cords were quantified as previously described. Briefly, spinal cord tissue was homogenized in 0.1 M perchloric acid to extract protein precipitates. Amino acids were derivatized with *o*-phthalaldehyde/2-mercaptoethanol and subsequently applied to liquid chromatography using an ODS-3 column (1.0×100 mm; GL Science, Tokyo, Japan) and fluorescence detection [46]. Protein was measured using a BCA protein assay reagent (Pierce, Rockford, IL, USA) using BSA as the standard.

### Electron Microscopy

Gluteus maximus muscles from mice at E18.5 were fixed in 3% (w/v) glutaraldehyde in 0.1 M phosphate buffer, pH 7.2 (PB) for 12 hours at 4°C. Fixed muscles were washed in PB, post fixed in 1% (w/v) aqueous osmium tetroxide containing 1.5% (w/v) potassium ferrocyanide [47], stained *en bloc* with uranyl acetate, dehydrated through acetone into Epon resin. Ultrathin sections were cut at ∼ 80 nm, stained with Reynolds lead citrate [48], viewed and photographed with a Hitachi H600 transmission electron microscope (Tokyo, Japan).

### Microscopy and Imaging

Sections were analyzed and images recorded using a Zeiss Axioplan 2 optical microscope coupled to a digital colour camera (Carl Zeiss, Gottingen, Germany). Images were saved in TIFF format and exported to Photoshop 7.0 (Adobe Systems Inc., CA, USA) for figure formatting. Brightness and contrast adjustments were made where applicable. All immuno-stained muscles were viewed using a Zeiss LSM 519 META scanning confocal microscope. A Z series of each muscle was collected and projected into a single image using NIH Image J image software [49]; (available from http://imagej.nih.gov/ij/).

### Data Analysis

At E13.5, E15.5 and E18.5 unpaired two tailed *t*-tests were conducted between the wild-type (GAD67^+/+^), and GAD67-deficient (GAD67^−/−^) mice for all analyses for each motor pool. Significance was set at *P*<0.05 using the Prism statistical program (GraphPad Software, San Diego, CA, USA).

## Results

### Decreased GABA Content within the Developing Spinal Cords of GAD67-deficient Mice

Previous studies have quantitated that GABA is decreased by approximately 80 to 90% in the cerebral hemispheres of GAD67 mutant mice during development [2], [34]. To check that GAD67 was also responsible for GABA synthesis within the developing spinal cord, we measured the amount of GABA in the cervical and lumbar regions of E18.5 spinal cords from GAD67-deficient mice compared to their wild type littermates. GABA content (mean ± SEM, nmol/mg protein) within the cervical portion of the spinal cord in GAD67- deficient mice (−/−) was only 17% of that of the wild type (+/+) control (−/−7.83±1.3; +/+47.39±2.03, *n* = 5, *P*<0.001). A similar decrease in GABA content was also observed in the lumbar region in mice lacking GAD67 (16% of that of the wild type control; −/−6.53±1.4; +/+39.92±2.08, *n* = 5; *P*<0.001). This data shows that the drop in GABA content within the developing spinal cord was reduced by the same amount to that previously reported for the brain in mice lacking GAD67 [2], [34].

### Regional Differences in Embryonic Motor Neuron Survival in GAD67-deficient Mice

To determine if there was a spatial and temporal regulation of embryonic motor neuron survival between respiratory-related and locomotor-related motor pools in mice lacking physiological levels of GABA, we examined four different regions of the brainstem and spinal cord at E13.5, E15.5 and E18.5. This represents the beginning, middle and end of developmental motor neuron cell death in the mouse [1], [3], [4], [5], [6], [7]. Hypoglossal and phrenic nuclei were considered to be respiratory-related motor nuclei as these motor neurons innervate the tongue and diaphragm respectively and are essential for proper breathing (e.g. [50], [51]). Brachial and lumbar lateral motor columns (LMCs) innervate forelimbs and hindlimbs respectively and were considered to be locomotor-related motor nuclei.

**Figure 2:**
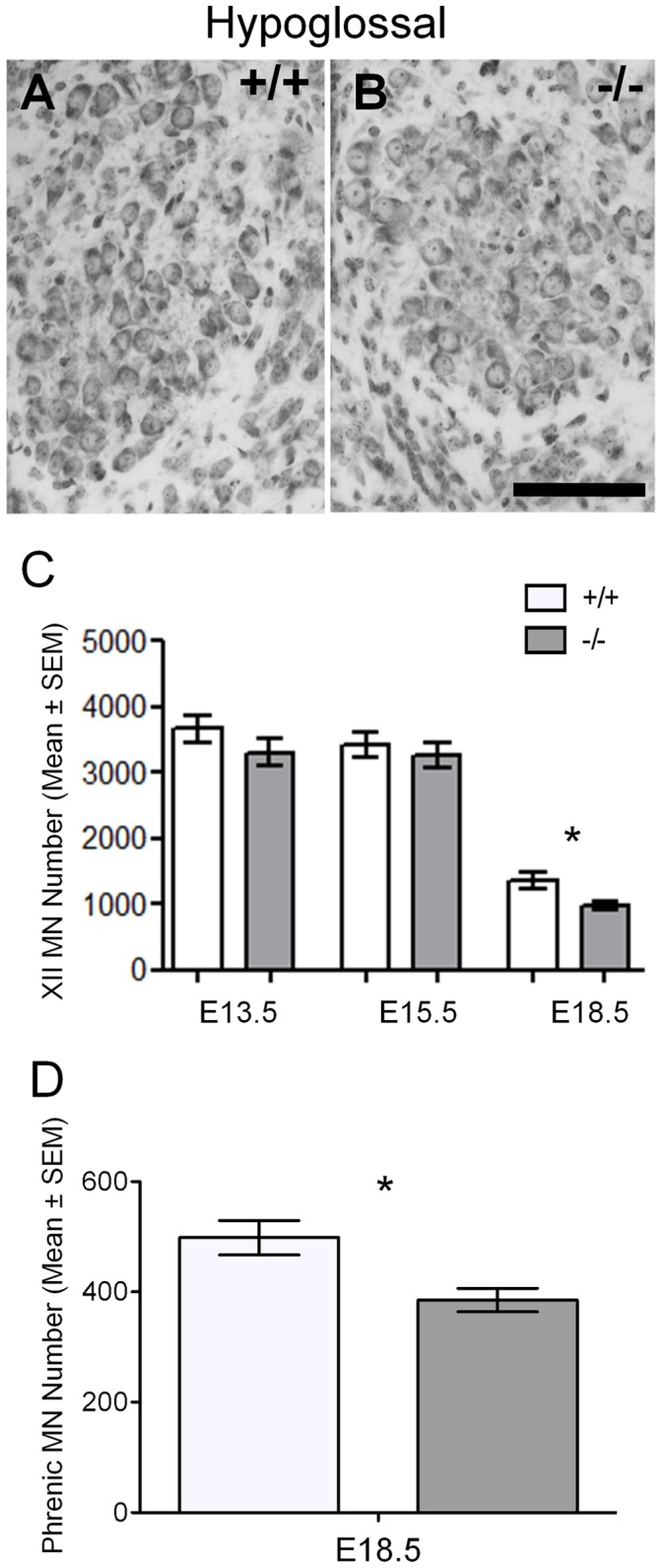
Decreased motor neuron survival in E18.5 GAD67-deficient (−/−) hypoglossal and phrenic motor nuclei. A and B show light micrographs of the hypoglossal (XII) motor nuclei from GAD67 deficient (B) and wild type littermate control (A) at E18.5. C and D show the mean motor neuron numbers ± SEM of hypoglossal motor neurons (C) from GAD67-deficient (−/−) and wild type (+/+) littermates respectively, from embryonic day 13.5 (E13.5) through to E18.5 and phrenic motor neurons numbers from GAD67-deficient (−/−) and wild type (+/+) littermates respectively at E18.5 only (D) (*n* = 6 per age, *P<0.05, unpaired *t* test). Scale Bar: A, B, 100 µm.

At the beginning and middle stages of the motor neuron death period, we observed no significant changes in motor neuron number between GAD67-deficient and wild type litter mate embryos for hypoglossal motor pools (Figure 2, Table 1); however by E18.5, we did observe significantly fewer motor neurons in the hypoglossal and phrenic motor pools in GAD67-deficient embryos compared to their wild type litter mates (Hypoglossal: 17% decrease; Phrenic: 23% decrease; Figure 2,Table 1). Thus, in embryos lacking physiological levels of GABA, fewer respiratory motor neurons were present by the end of the developmental neuronal death period.

**Table 1:** Motor neuron numbers are decreased in respiratory motor nuclei (hypoglossal, phrenic) and increased in forelimb innervating motor nuclei (brachial) in GAD67-deficient mice (−/−) compared to wild-type littermate mice (+/+).

Motor Nucleus	+/+	−/−	% Change (−/−relative to +/+)	*P* value
Hypoglossal (XII)				
E13.5	3669±205 (4)	3310±202 (4)	−9.8%	0.2579
E15.5	3428±193 (6)	3263±192 (6)	−4.8%	0.5574
E18.5	1362±119 (6)	989±63 (6)	−17.4%	* 0.02
Phrenic (C3–5)				
E18.5	498±31 (3)	385±21 (3)	−22.7%	* 0.0386
Brachial (C6–8)				
E13.5	5651±267 (6)	5281±310 (6)	−6.5%	0.3868
E15.5	3343±116 (6)	4164±158 (6)	24.6%	** 0.0018
E18.5	2141±111 (5)	3053±264 (5)	43%	** 0.009
Lumbar (L2–6)				
E13.5	4506±232 (5)	4673±118 (4)	3.7%	0.5742
E15.5	3226±314 (6)	3385±181 (6)	4.9%	0.6705
E18.5	2175±99 (6)	2193±142 (6)	−5.6%	0.9222

No influence on survival of hindlimb innervating motor nuclei (lumbar).

Values represent the mean number of motor neurons ± SEM. Values in parentheses indicate the number of animals examined.

* *P*<0.05

** *P*<0.01; unpaired two tailed Student’s *t*-test.

**Figure 3:**
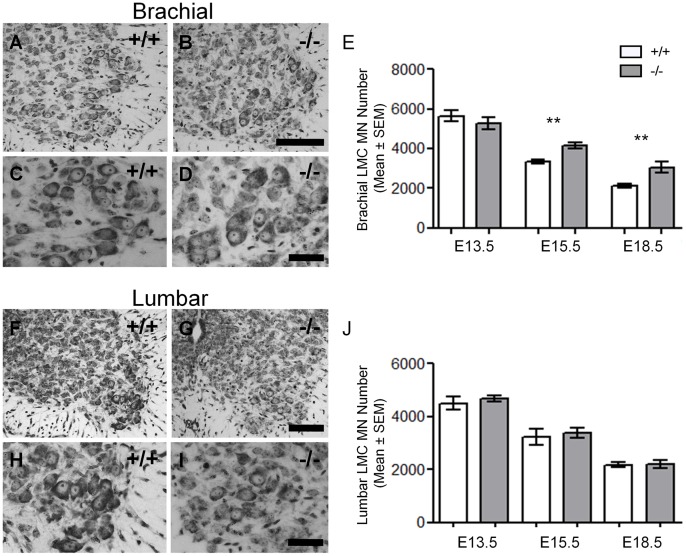
Increased motor neuron survival in E15.5 and E18.5 GAD67-deficient (−/−) brachial motor nuclei. A to D and F to I show light micrographs of the brachial and lumbar motor nuclei from GAD67-deficient (B, D, G, I) and wild type littermate control (A, C, F, H) at E18.5. C-D and H-I show motor neurons within the brachial and lumbar lateral motor columns at higher magnification, respectively. E and J show the mean motor neuron numbers ± SEM of brachial motor neurons (E) and lumbar motor neurons (J) from GAD67-deficient (−/−) and wild type (+/+) littermates respectively, from embryonic day 13.5 (E13.5) through to E18.5. Increased motor neuron survival was observed in GAD67-deficient mice for brachial motor LMC nuclei (E), whereas motor neuron numbers were unchanged for lumbar motor LMC nuclei (J), compared to wild type (*n* = 6 per age, **P<0.01, unpaired *t* test). Scale Bar: A, B, F, G, 100 µm, C, D, H, I, 50 µm.

As inhibitory neurotransmission also occurs on locomotor motor neurons in the brachial and lumbar spinal cord, motor neuron survival counts were performed at E13.5, E15.5 and E18.5 in wild type and GAD67-deficient mice. As seen for the hypoglossal respiratory motor pool, we observed no significant change in the number of brachial or lumbar LMC motor neurons between GAD67-deficient and their wild type littermates at E13.5 (Table 1, Figure 3). By the middle stage of motor neuron cell death (E15.5) when synapses are forming onto and by motor neurons, we observed an increase in brachial LMC motor neuron survival in GAD67-deficient mice compared to their wild type littermates (25% increase; Table 1, Figure 3). This difference in brachial LMC motor neuron survival persisted throughout the period of motor neuron cell death (E18.5∶43% increase; Table 1, Figure 3). By contrast, we observed no significant differences in motor neuron survival for lumbar LMC motor neurons throughout the entire motor neuron death period (E13.5 to E18.5; Table 1; Figure 3). Thus, in embryos lacking physiological levels of GABA, only brachial LMC motor neuron survival was affected, whereas for more caudally located lumbar LMC motor neurons, a marked reduction in endogenous GABA had no effect on motor neuron survival.

### No Changes in Regional and Motor Neuron Morphology in Mice Lacking GABA

**Figure 4:**
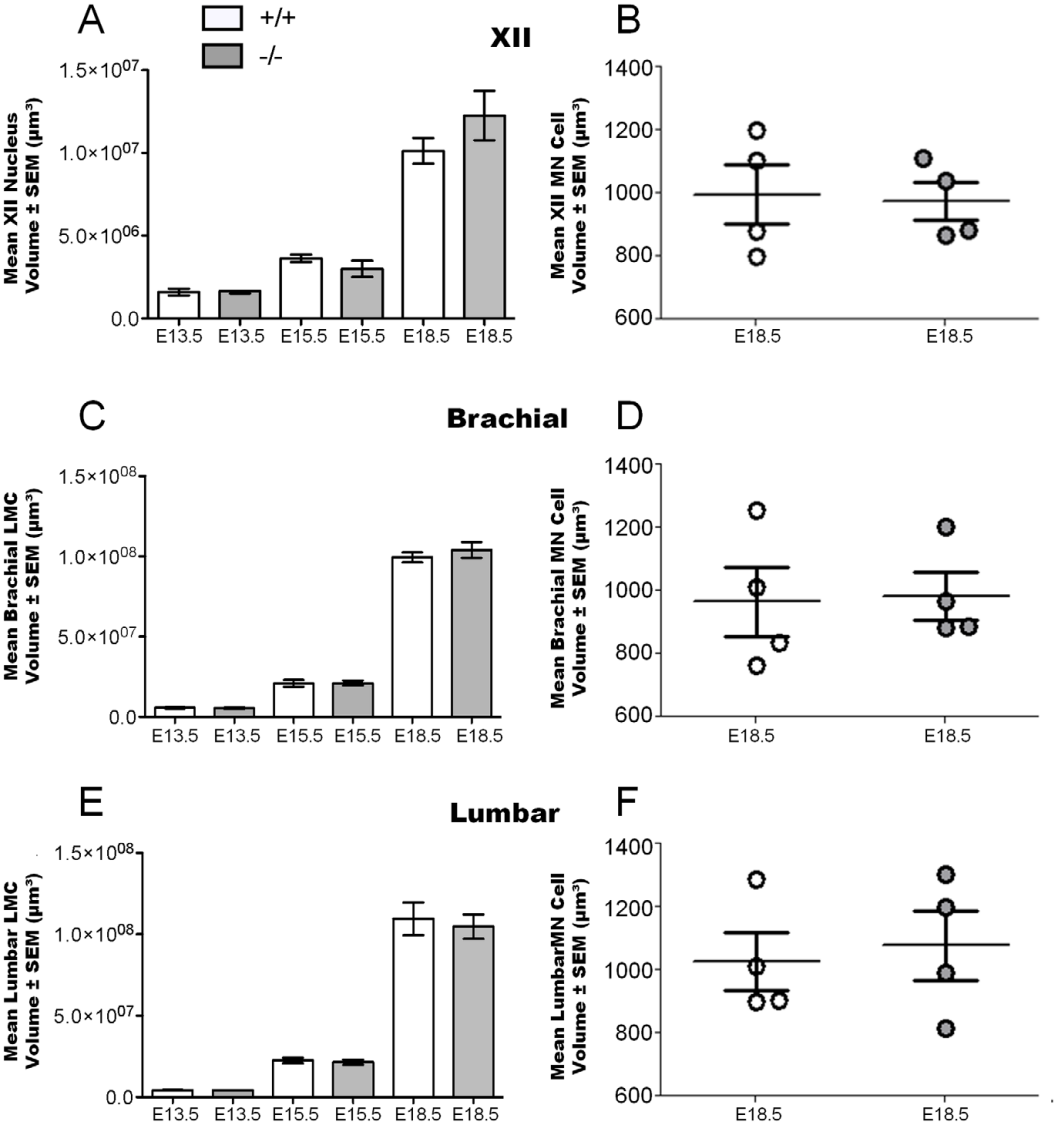
Motor neuron and spinal cord morphology are unchanged in GAD67 deficient (−/−) mice. (**A**) Cavalieri volume estimation of the mean hypoglossal (XII) motor nucleus volume ± SEM (*n* = 3 for E13.5 and E15.5, *n* = 4 for E18.5, *P*>0.05, unpaired *t* test). (**B**) Estimation of XII motor neuron cell body volume ± SEM (*n* = 4, *P*>0.05, unpaired *t* test). (**C**) Cavalieri volume estimation of the mean brachial lateral motor column volume ± SEM (*n* = 3 for E13.5, *n* = 4 for E15.5 and E18.5, *P*>0.05, unpaired *t* test). (**D**) Estimation of brachial motor neuron cell body volume ± SEM (*n* = 4, *P*>0.05, unpaired *t* test). (**E**) Cavalieri volume estimation of the mean lumbar lateral motor column volume ± SEM (*n* = 4 for all ages, *P*>0.05, unpaired *t* test). (**F**) Estimation of lumbar motor neuron cell body volume ± SEM (*n* = 4, *P*>0.05, unpaired *t* test).

To assess if reduced levels of GABA would alter the regional appearance of the spinal cord (i.e. sizes and location of the motor nuclei/pool) and motor neuron morphology, we quantified motor pool volume as well as the size of individual motor neurons within these motor pools in GAD67-deficient and wild type littermates. We found no differences in the gross appearance, size and location for hypoglossal motor nuclei, brachial or lumbar LMCs in GAD67-deficient mice compared to wild type littermates, at all stages studied (Figure 2A and B for hypoglossal; Figure 3A to D for Brachial LMC; Figure 3 F to I for Lumbar LMC; and Figure 4A, C and E for motor pool volumes). Hence, the deficit in GABA did not affect gross development of the brain stem and spinal cord.

Next we measured the cell soma size (volume) of individual motor neurons within these motor pools, using stereological techniques (see methods for details). Motor neuron volume was not altered in any motor pool in GAD67-deficient mice compared to wild type littermates, at E18.5 (Figure 4B, D and F). To ensure the veracity of these measurements, quantification of all cell soma volumes within these motor pools was done at E18.5. We observed no shift in the distribution of large or small cells across the two genotypes; namely the cell volume frequencies of small and large cells did not change between GAD67-deficient and wild type mice (Figure 1A). We have defined these large cells as motor neurons, based upon accepted histological identification criteria ( [5], [36]; see methods), and also supported by our cell size comparisons of thionin and VAChT stained cells (Figure 1B and C). Hence, the deficit in GABA did not affect the size distributions of all cells including motor neurons within the studied motor regions.

### Muscle-nerve Branching and Neuromuscular Synapse Number are Altered in GAD67-deficient Mice, Except for Lumbar Motor Neurons

Previous research has shown that decreased motor neuron survival correlated with a decrease in both muscle-nerve branching and neuromuscular synapse number [1], [11], [52] and vice versa [1], [5], [7], [8], [9], [10], [53]. Hence, we wanted to know if the regional changes in motor neuron survival seen in GAD67-deficient mice (Table 1) resulted in corresponding changes in muscle-nerve branching and neuromuscular synapse number in respiratory (diaphragm) and limb muscles at E15.5 (mid period of motor neuron death, and early stages of neuromuscular synapse formation) and at E18.5 (late stage of motor neuron death).

**Figure 5:**
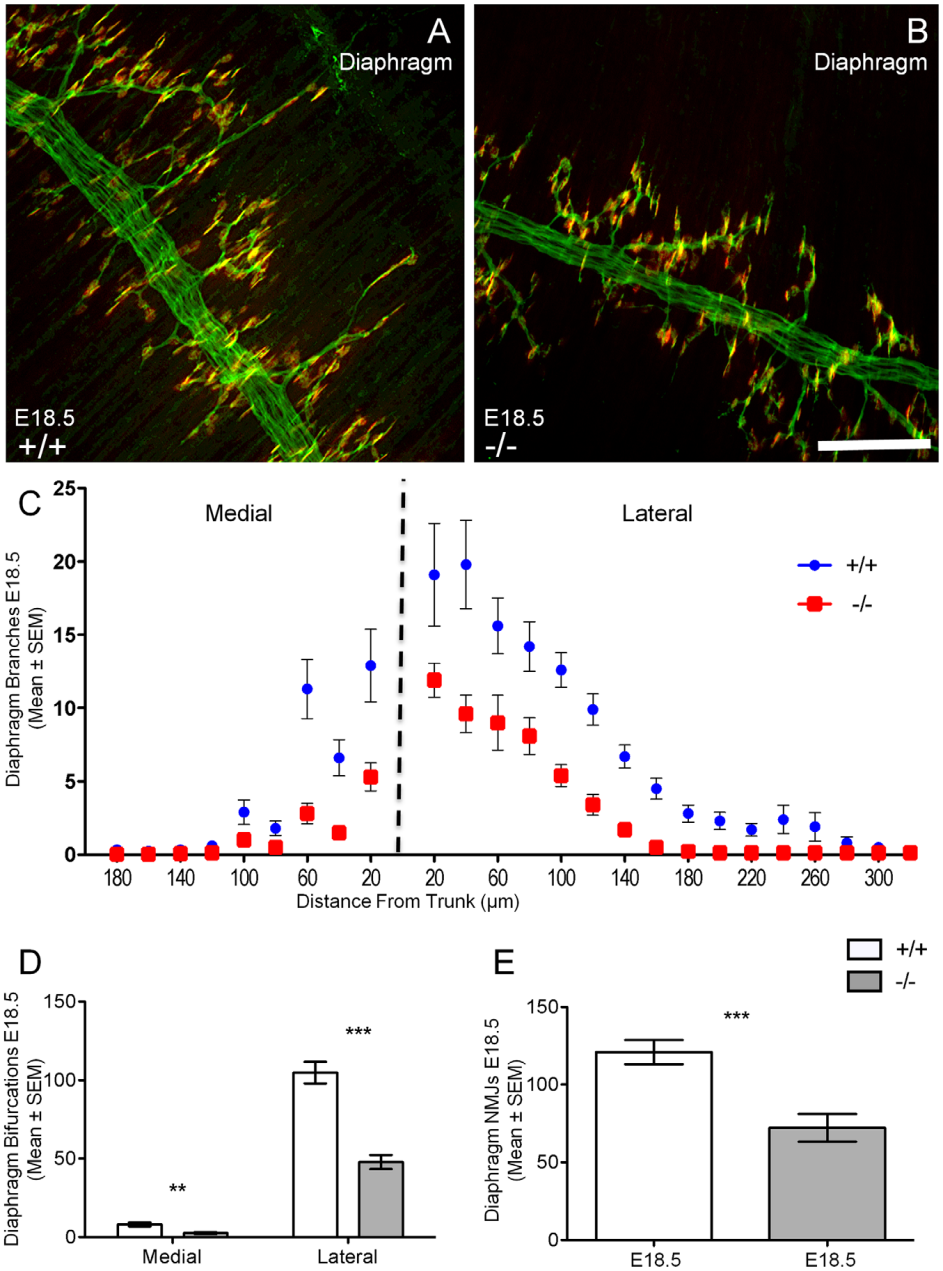
Decreased diaphragm muscle axonal branching distance, bifurcations and neuromuscular junctions in GAD67-deficient (−/−) E18.5 mice. Axonal branches (green) and acetylcholine receptor clusters (red) in the diaphragm muscle in E18.5 wild type (A, *+/+*) and GAD67-deficient (B, *−/−*) are shown. C and D show significant decreases in the number of medial and lateral axonal branches (mean ± SEM) respectively at discrete distances away from main nerve trunk in GAD67-deficient (red) mice compared to wild type controls (blue). D shows a significantly decreased medial and lateral bifurcation number (mean ± SEM) in GAD67-deficient mice compared to wild type (*n* = 10, **P<0.01, ***P<0.001, unpaired *t* test). E shows a significant decrease in the number of neuromuscular junction endplates in the diaphragm of GAD67-deficient compared to wild type littermates. (*n* = 10, ***P<0.001, unpaired *t* test). Scale Bar: A, B, 100 µm.

**Figure 6:**
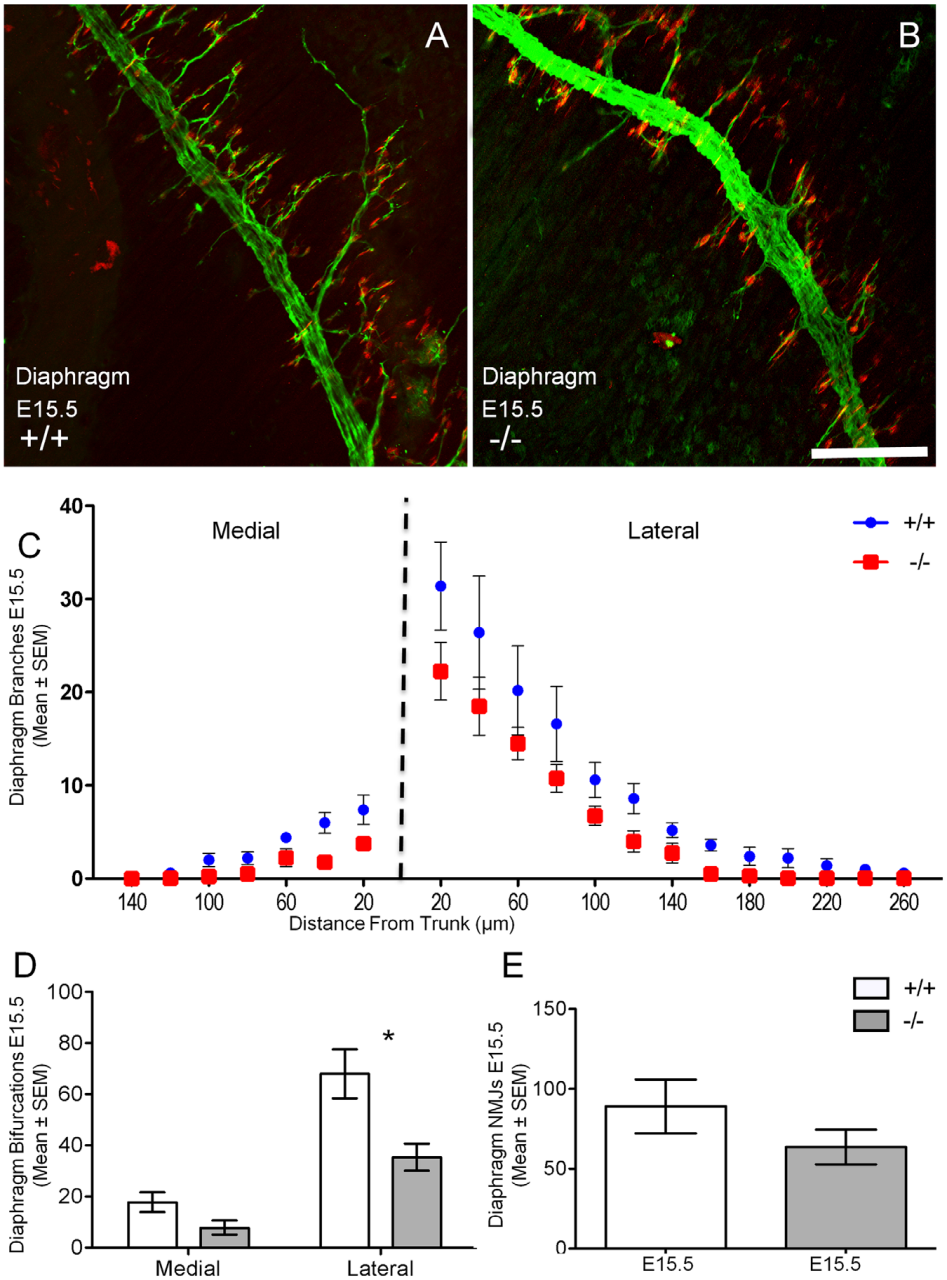
Decreased diaphragm muscle lateral bifurcations in GAD67-deficient (−/−) E15.5 mice. Axonal branches (green) and acetylcholine receptor clusters (red) in the diaphragm muscle in E15.5 wild type (**A**, *+/+*) and GAD67-deficient (**B**, *−/−*) are shown. **C** and **D** show a significant decrease in the number of lateral axonal branches (mean ± SEM) respectively at discrete distances away from main nerve trunk in GAD67-deficient (red) mice compared to wild type controls (blue). **D** shows a significantly decreased lateral bifurcation number but not medial bifurcation (mean ± SEM) in GAD67-deficient mice compared to wild type (*n* = 5, *P<0.05, unpaired *t* test). **E** shows no change in the number of neuromuscular junction endplates in the diaphragm of GAD67-deficient mice compared to wild type littermates (*n* = 5, *P*>0.05, unpaired *t* test). Scale Bar: **A**, **B**, 100 µm.

At E18.5 and E15.5, diaphragm muscle nerve branching and neuromuscular location show little variation in GAD67-deficient and wild type mice [1], [7]. The phrenic nerve trunk within the ventral sterno-costal portion of the diaphragm ran perpendicular to muscle fibers and was restricted to the midline of the muscle in the last 1.2 mm before the muscle insertion (Figures 5 and 6). Intra-muscular axonal branches extended primarily toward the lateral side of the phrenic nerve trunk in both wild type (Figures 5A and 6A) and GAD67-deficient (Figures 5B and 6B) diaphragms. However, both medial and lateral intra-muscular axons showed significant reductions in the distance they extended from the phrenic nerve trunk in comparison to their wild type littermates (Figure 5A, B and C), at E18.5. This same trend, albeit to a lesser extent was also apparent at E15.5, (Figure 6A, B and C) during the early stages of diaphragm innervation [54].

We next quantified the number of intramuscular axonal bifurcations (i.e. branching) within the diaphragms of E18.5 and E15.5 wild type and GAD67-deficient mice. At E18.5, the number of medial and lateral axonal bifurcations (mean ± SEM) was significantly decreased for the GAD67-deficient (medial, 33% decrease, 2.7±0.67, *P*<0.01; lateral, 55% decrease, 47.8±4.53, *P*<0.001, *n* = 10) diaphragm muscle in comparison to wild-type littermates (medial, 8.1±1.25; lateral, 104.7±6.94, *n* = 10; Figure 5D). At E15.5 we observed a similar degree of decreased lateral bifurcations of GAD67-deficient (−/−) mice compared to wild type (+/+) littermates (*−/−* lateral, 52% decrease, 35.4±6.27; *+/+* lateral, 68±9.55; *P*<0.05, *n* = 6; Figure 6D). We then counted neuromuscular junctions within the ventral portion of the left hemi-diaphragm at E15.5 and E18.5, and found no change when comparing GAD67-deficient diaphragms to their wild type littermates at the early stages of neuromuscular synapse formation (E15.5, Figure 6E). However by E18.5, the number of neuromuscular junctions within these muscle regions had significantly decreased in the GAD67-deficient (40% decrease, 72.2±8.88, *P*<0.001, *n* = 10) diaphragm muscle in comparison to their wild-type littermates (121.0±7.80, *n* = 10; Figure 5E). Thus, the significant decrease in phrenic motor neuron survival correlated with a significant decrease in diaphragm innervation in mice lacking physiological levels of GABA, at E18.5.

**Figure 7:**
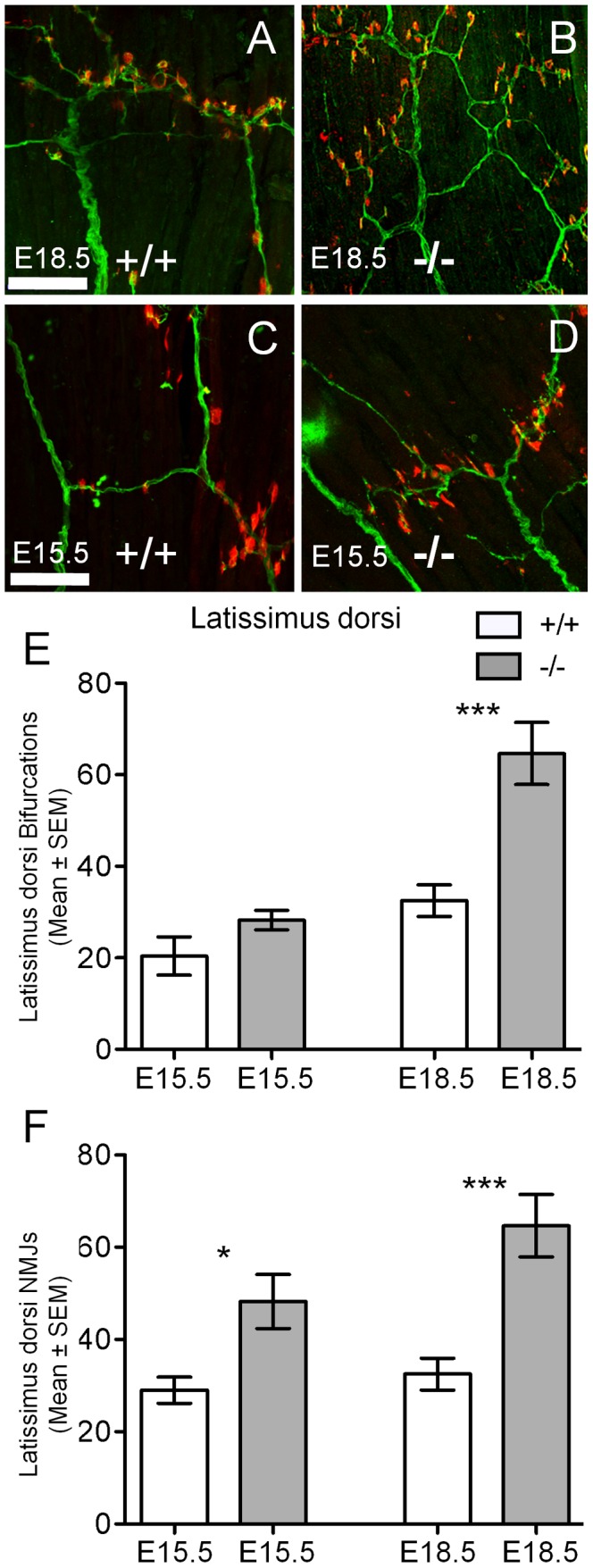
Increased latissimus dorsi axonal branching bifurcations and neuromuscular junctions in GAD67-deficient (−/−) mice. Axonal branches (green) and acetylcholine receptor clusters (red) in the latissimus dorsi muscle in E18.5 wild type (**A**, *+/+*) and GAD67-deficient (**B**, *−/−*), and E15.5 wild type (**C**) and GAD67-deficient (**D**). (**E**) shows a significantly increased latissimus dorsi nerve bifurcation number (mean ± SEM) in GAD67-deficient mice compared to wild type at E18.5 (*n* = 4 for E15.5, *n* = 8 for E18.5, ***P<0.001, unpaired *t* test). (**F**) shows increased number of latissimus dorsi neuromuscular junction endplates (mean ± SEM) in the GAD67-deficient compared to wild type. (*n* = 4 for E15.5, *n* = 8 for E18.5, *P<0.05, ***P<0.001, unpaired *t* test). Scale Bar: **A**, **B**, **C**, **D**, 75 µm.

**Figure 8:**
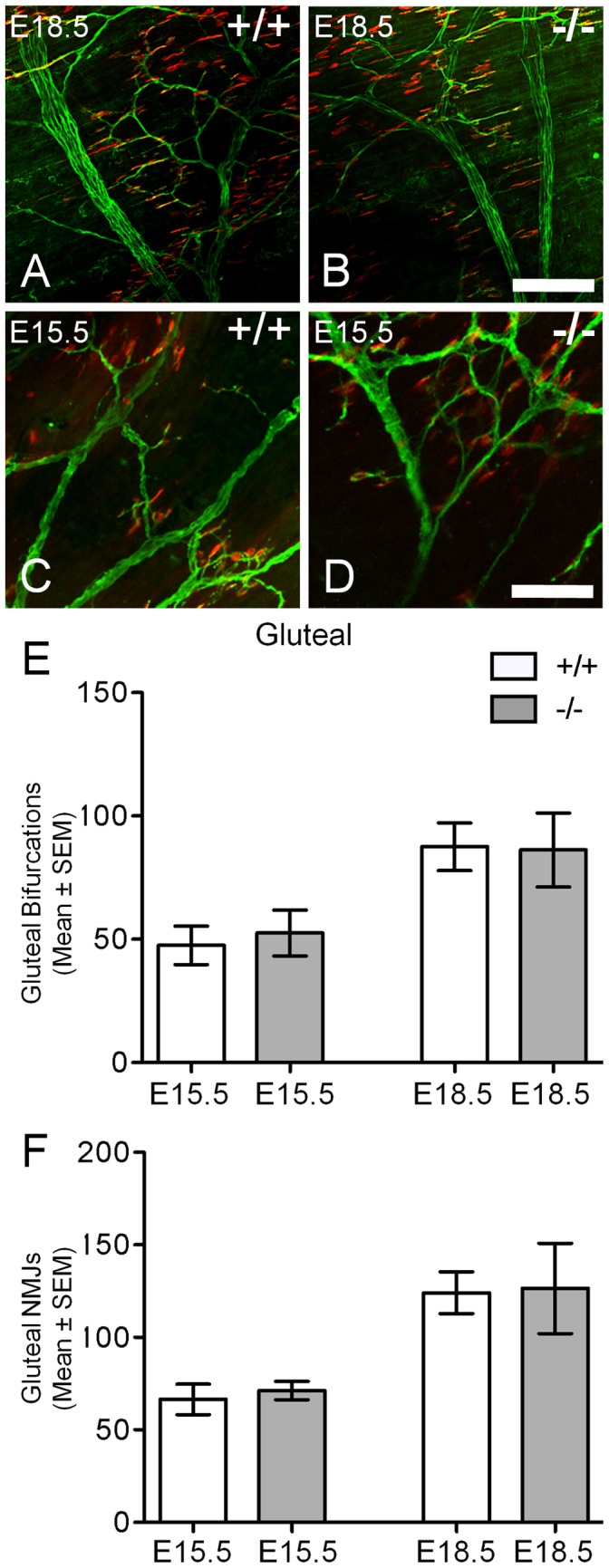
Unchanged gluteus maximus axonal branching bifurcations and neuromuscular junctions in E15.5 or E18.5 GAD67-deficient (−/−) mice. Axonal branches (green) and acetylcholine receptor clusters (red) in the gluteus maximus muscle in E18.5 wild type (A, *+/+*) and GAD67-deficient (B, *−/−*) and E15.5 wild type (C) and GAD67-deficient (D) mice. (E) shows no differences in gluteus maximus nerve bifurcation number (mean ± SEM) in GAD67-deficient mice compared to wild type (*n* = 4 for E15.5, *n* = 6 for E18.5, *P*>0.05, unpaired *t* test). (F) shows no change in the number of gluteus maximus neuromuscular junction endplates (mean ± SEM) in the GAD67-deficient mice compared to wild type littermates (*n* = 4 for E15.5, *n* = 6 for E18.5, *P>0.05, unpaired *t* test). Scale Bar: A, B, C, D, 75 µm.

To determine if reduced levels of GABA neurotransmitter within the spinal cord would alter the innervation of fore and hind limb muscles, we also examined their innervation patterns (i.e. muscle-nerve branching and neuromuscular synapse number) in the latissimus dorsi and gluteus maximus muscles. These muscles are innervated by motor neurons originating from brachial and lumbar LMCs respectively [38], [55]. Within the latissimus dorsi, the number of axonal bifurcations was significantly increased in GAD67-deficient (99% increase, 64.67±6.79, *P*<0.001, *n* = 6; Figure 7B and 7E) in comparison to wild-type littermates (32.50±3.48, *n* = 8; Figure 7A, and 7E) at E18.5. Closer inspection of these stained muscles revealed that its muscle nerve was highly branched, with increased numbers of neuromuscular synapses over the same muscle portion in GAD67-deficient (−/−) compared to their wild type (+/+) littermates (−/−, 116% increase, 82.67±6.84, *n* = 6; *+/+*38.25±3.72, *n* = 8, *P*<0.0001; Figure 7A, B, and F). During the earlier stages of neuromuscular synapse formation in this muscle (E15.5), the extent of increased muscle nerve branching in the GAD67-deficient mice was not significantly different to that of their wild type littermates (Figure 7C, D and E). However, we did observe a significant increase in the number of neuromuscular synapses within the same muscle regions at this stage (−/−, 66% increase, 48.25±5.85, *n* = 4; *+/+*29±2.83, *n* = 5, *P*<0.05; Figure 7F). By contrast, when we examined the innervation of the gluteus maximus muscle supplied by motor neurons of the lumbar LMC, we observed no change in axonal branching bifurcations or in the numbers of neuromuscular synapses within the analyzed region in GAD67-deficeint mice compared to their wild type littermates (Figure 8). Thus, the significant increase in brachial motor neuron survival correlated with a significant increase in forelimb latissimus dorsi muscle innervation in mice lacking physiological levels of GABA. Whereas for lumbar LMC motor neurons, we observed no significant changes in hindlimb gluteus maximus muscle innervation, which correlated with no changes in lumbar motor neuron survival, at all stages studied.

**Figure 9:**
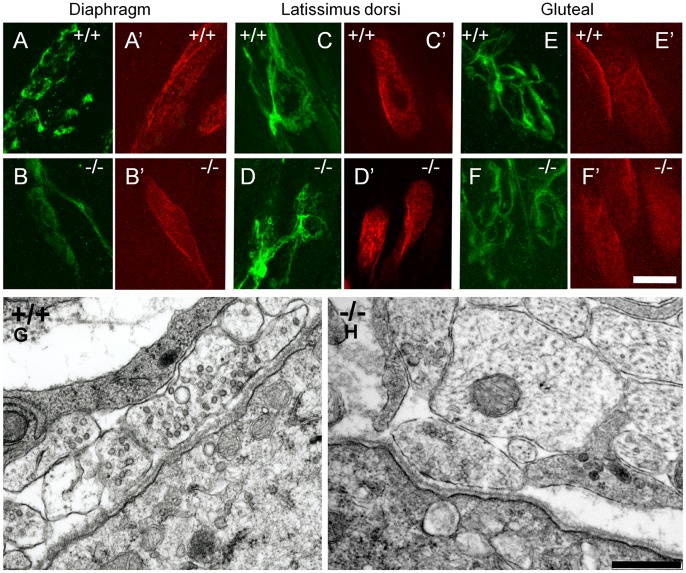
Development and ultrastructure of neuromuscular junction endplates is not affected by GAD67 deficiency at E18.5. Neuromuscular junction endplates stained with anti-neurofilament plus anti-synaptophysin (green) and for muscle acetylcholine receptors with α-bungarotoxin (red). **A** and **A’** show E18.5 wild type diaphragm neuromuscular junction endplates compared to GAD67-deficient (−/−) mice in **B** and **B’**. **C** and **C’** show E18.5 wild type latissimus dorsi neuromuscular junction endplates compared to GAD67-deficient (−/−) mice in **D** and **D’**. **E** and **E’** show E18.5 wild type gluteal neuromuscular junction endplates compared to GAD67-deficient (−/−) mice in **F** and **F’**. Electron micrographs of single neuromuscular junction endplates in the gluteal muscle show no ultrastructural differences between wild type (**G**) and GAD67-deficient (**H**) mice. Scale Bar: **A**, **A’**, **B, B’, C, C’, D, D’, E, E’**, **F**, **F’,** 20 µm; **G**, **H**, 500 nm.

We also examined the morphology of neuromuscular junctions (NMJs) from diaphragm, latissimus dorsi and gluteus maximus muscles from GAD67-deficient and wild type littermates at E18.5. This was done using confocal and electron microscopy. Under confocal fluorescence we observed no qualitative change in the overall size of the NMJ, nerve terminal endings, or in the appearance of post-synaptic acetylcholine receptor clusters in these muscles from GAD67-deficient mice compared to their wild type littermates, at E18.5 (Figure 9A to F; NMJs observed across 3 wild type and 3 GAD67-deficient mice). These observations were supported by electron microscopy, where we observed no obvious qualitative change in the appearance of motor nerve terminal endings, synaptic cleft, and synaptic basal lamina organization of NMJs from GAD67-deficient and wild type mice (Figure 9G and H).

## Discussion

Our study reveals a role for GABA in motor neuron development and muscle innervation. Mice lacking physiological levels of GABA showed regionally distinct changes in motor neuron number and muscle innervation, during the period of naturally occurring motor neuron death (E13.5 to E18.5). Respiratory motor neurons showed decreased motor neuron survival and restricted innervation patterns. By contrast, brachial LMC motor neurons (locomotor motor neurons) displayed increased motor neuron survival and expanded innervation patterns, whereas we observed no changes in lumbar LMC motor neuron survival and innervation of hind limb muscle. These findings are similar to what we have reported for mice that have perturbed glycinergic transmission, except that in gephyrin-deficient mice we did see increased lumbar LMC motor neuron number and expanded hind limb muscle innervation [1]. Together our results suggest that GABA and glycine make similar contributions to neuromotor development for respiratory and brachial LMC motor neurons and their muscle innervations, but for more caudal (lumbar) regions of the spinal cord, GABA has little effect on motor neuron development, compared to that of glycine. The implications of these findings are discussed below.

### GABA Regulates Motor Neuron Survival and Innervation According to Region and Physiological Function

The observed increases in brachial motor neuron survival and innervation of the latissimus dorsi muscle are consistent with previous genetic and pharmacological perturbations that have identified that the level of neuromuscular synaptic activity, which determines the level of muscle activity, is a critical regulator of motor neuron development and innervation. Many previous studies have demonstrated that lower levels of motor neuron activity, or perturbations to neuromuscular function (transmission or formation) correlates with increased motor neuron survival, muscle-nerve branching and neuromuscular synapse number [1], [5], [7], [8], [9], [10], [11], [53], [56], [57], [58], [59], [60], [61], [62], [63], [64], [65]. The converse response is also true, namely, that increased motor neuron activity leading to increased muscle activity via intact neuromuscular synapses or electrical overstimulation of skeletal muscle, results in increased motor neuron death and restrictions in muscle innervation [1], [11]. Our results for respiratory (hypoglossal and phrenic) motor neurons are also consistent with this relationship between motor neuron activity, motor neuron survival and muscle innervation. Taken together, our results suggest that for respiratory and non-respiratory brachial motor neurons, GABA may play a similar role to what we have reported for glycine in the regulation of motor neuron development and innervation for these motor pools [1].

In our previous study [1], we have shown that spontaneous motor neuron activity is higher in lumbar (L5) motor neurons in comparison to respiratory (hypoglossal) motor neurons at birth. Upon perturbation of glycinergic transmission, these levels of motor neuron activity are reversed (i.e. respiratory motor neuron activity is increased, and lumbar motor neuron activity is decreased), resulting in decreased respiratory motor neuron survival and restricted innervation of the diaphragm, and the opposite responses for lumbar motor neurons and innervation of the gluteus maximus muscle. These and our present results for respiratory and brachial motor neurons, would suggest that GABA has switched to being an inhibitory neurotransmitter for respiratory motor neurons early in development, whereas our data suggests that GABA has remained excitatory at E18.5 for non-respiratory (brachial) motor neurons. Support for these different GABAergic functions comes from previous work, where glycine and GABA_A_ receptor activation increases lumbar motor activity in embryonic mouse [17], and rat [23], [66], switching to an inhibitory function (decreased activity) just prior to birth [23], [67]. By contrast, glycine and GABA_A_ receptor activation depresses respiratory motor frequency soon after the formation of neuromuscular synapses, from E16 onwards [68]. We propose that GABA may act to differentially regulate motor neuron activity, which in turn regulates the level of muscle activity through intact innervation, because neuromuscular junctions develop normally in mice lacking physiological levels of GABA (GAD67-deficient mice). Hence our data supports the idea that regulation of motor pool size (i.e. number of motor neurons dedicated to a specific anatomical muscle), is dependent upon the emerging levels of motor neuron activity that is in turn passed onto muscle during development.

If the level of muscle activity passed onto the innervating motor neuron acts to retrogradely regulate the muscle motor pool size and innervation, how might this be mediated? Past studies have proposed two possible mechanisms. The “production hypothesis” relies on the activity-dependent production of trophic and/or axon growth-adhesion promoting factors by the target muscle [8], [11], [52], [69], while the “access hypothesis” proposes that the level of muscle activity regulates the ability of motor neurons to access such muscle-derived factors through appropriate adjustment of neuromuscular synapse numbers [8], [62], [69], [70], [71]. It is possible for both mechanisms to be operating simultaneously, with muscle activity being the key to limiting both production and access of such factors to the post-synaptic region of the muscle in a way that is similar to the mechanisms proposed to restrict acetylcholine receptor gene expression to the nuclei directly beneath the motor nerve terminal (reviewed by [72], [73]). While our study was not designed to investigate these possible mechanisms, our observations are consistent with them, in that restricted muscle innervation and numbers of motor neurons would be consistent with increased muscle activity and a restricted production and thereby limited availability of possible neurotrophic and/or axon growth-adhesion factors to the post-synaptic region.

While we favour this interpretation, it is also possible that a lack of GABA has affected the development of these motor neurons in ways other than its direct role as a neurotransmitter [74], [75]. This is plausible, as GABA’s receptors include both chloride-permeable ligand gated ion channels (GABA_A_ and GABA_C_ receptors) and G-protein coupled receptors (GABA_B_ receptors), all of which can be expressed by motor neurons [76], [77], [78], [79]. Hence activation of GABA’s receptors by GABA will not only trigger chloride ion-dependent synaptic transmission, but a number of other signaling mechanisms that could potentially affect motor neuron development, including motor activity [75]. For example, previous studies have shown that GABA acting via its receptors can affect the migration and differentiation of neuronal precursors in the developing brain [80], [81], [82]; maturation of hippocampal neurons [83]; ability to regulate the release of neurotropic factors such as BDNF, a potent motor neuron trophic factor from cortical neurons [84], promotion of synapse formation in the hippocampus [81], [85], and glial cell activation which in turn could aid in glia mediated neuronal differentiation and transmission [86].

### GABA does not Appear to Regulate Lumbar Motor Neuron Development and Innervation

In mice lacking physiological levels of GABA (GAD67-deficient mice), we observed no changes in lumbar LMC motor neuron number and no changes in their hind limb innervation patterns. These findings are in contrast to our previous studies in mutant mice where glycinergic transmission has been disrupted (gephyrin deficient mice), where we saw increased lumbar LMC motor neuron number and innervation of hind limb muscles [1]. Our simplest interpretation is that GABA has little influence on motor neuron development and muscle innervation for these caudal lumbar motor neuron pools, in that glycine is able to effectively compensate for a loss of GABA. In support of this, recent studies have determined the relative densities of GABA and glycine containing neurons within the ventral regions of the developing mouse spinal cord (E11.5 through to E17.5-birth; [87], [88]). Throughout the course of developmental motor neuron death period (E12.5 to E17.5-birth), the density of GABAergic neurons within the lumbar spinal cord (ventral regions) declines by 80% (from approximately 50,000 neurons per mm^3^ at E13.5 to 10,000 neurons per mm^3^ at P0), whereas the number of glycinergic neurons in the ventral horn doubles over the same period (from a numerical density of 26,000 to 57,000) [87], [88]. By contrast for brachial levels of the ventral spinal cord, the density of GABAergic neurons is higher at E13.5, decreasing by 80% at E15–17.5 ([87], [88]; i.e. from approximately 80, 000 neurons per mm^3^ at E13.5 to 15,000 neurons per mm^3^ at birth). The density of glycinergic neurons in the brachial ventral region also declines (from a numerical density of 80,000 at E13.5 to 54,000 at P0) during this same period, with 30% of the remaining cells being both GABAergic and glycinergic populations [88]. Taken together, this data suggests that glycinergic transmission in the lumbar cord can compensate or override the effects of GABAergic transmission in the absence of GABA; whereas for brachial regions, GABA plays a significant role in motor neuron survival, which cannot be compensated for by glycine in the absence of GABA.

This neuroanatomical data complements and reinforces functional data, which shows a progressive dominance of glycine and glycinergic transmission during the development of the embryonic mouse spinal cord [89], [90]. For lumbar motor neuron pools, these findings add support to our suggestion that the influence of GABA on motor neuron survival is minor, compared to that of glycine. It also supports the findings of our previous study in mice lacking gephyrin, a molecule that clusters post-synaptic glycine and some GABA_A_ receptors [30]. In that study, we showed that defective glycinergic transmission does alter lumbar motor neuron activity, survival and innervation, demonstrating that glycinergic transmission is having an effect on lumbar neuron motor pools [1].

In conclusion, we demonstrate here that GABA does make a contribution to motor neuron survival and muscle innervation during the period of naturally occurring cell death in mice/mammals, but that its influence is more restricted in comparison to that of glycine.
